# Noncontact Visualization of Respiration and Vital Sign Monitoring Using a Single Mid-Wave Infrared Thermal Camera: Preliminary Proof-of-Concept

**DOI:** 10.3390/s26010098

**Published:** 2025-12-23

**Authors:** Takashi Suzuki

**Affiliations:** Smart Life Science Laboratory, Center for Health Science Innovation, Osaka Metropolitan University, 1-4-3 Asahi-Machi, Abeno-ku, Osaka 545-8585, Japan; tszk@omu.ac.jp; Tel.: +81-6-6684-9356

**Keywords:** breath visualization, mid-wave infrared camera, carbon dioxide, vital signs, remote sensing

## Abstract

Infrared thermal cameras can noninvasively measure the surface temperatures of objects and are widely used as fever-screening systems for infectious diseases. However, body temperature measurements alone are often insufficient for identifying people with infections. To address the inherent limitations of fever-based screening, this study aimed to develop analytical methods that enable multi-vital sensing alongside body temperature measurement using a single mid-wave infrared (MWIR) camera. Respiratory parameters were assessed by visualizing exhaled airflow based on MWIR absorption by carbon dioxide, whereas the heart rate was estimated from subtle temperature fluctuations captured using high thermal resolution. The experimental results validated the proposed method, showing that the developed system achieved good agreement with reference measurements; the respiratory rate, heart rate, and body temperature showed strong correlations (r = 0.864–0.987) and acceptable limits of agreement in Bland–Altman analyses. The exhalation volume was quantified from the visualized airflow and was found to align with the expected physiological ranges. These results demonstrate that noncontact multi-vital sensing can be achieved using a single MWIR camera, without the need for complex instrumentation. The proposed method holds promise for high-precision infection screening, remote health monitoring, and in-home physiological assessment.

## 1. Introduction

Infrared thermal cameras have been widely used as screening tools for certain infectious diseases, such as coronavirus disease 2019 (COVID-19). Specifically, infrared thermal imaging is effective for the contactless measurement of elevated body temperature, enabling the screening of individuals with fever during an infectious disease epidemic or pandemic. However, relying solely on thermography to monitor and measure body surface temperature is insufficient for disease screening, primarily because individuals with infections may not exhibit fever during the incubation period. Notably, a high proportion of patients with COVID-19 did not experience fever [1,2,3]. Moreover, facial temperature can be affected by various factors, such as antipyretic consumption, alcohol consumption, cosmetics, pregnancy, and physical activity [4,5], leading to false-negative results and compromising the accuracy of temperature measurements when relying solely on thermography. Therefore, additional physiological states must be evaluated simultaneously to increase the accuracy of screening for infectious diseases.

In the clinical management of COVID-19, the respiratory rate, heart rate, and oxygen saturation should be monitored. Respiratory and heart rates are used to identify serious respiratory illnesses [6,7]. COVID-19 not only affects body temperature but also induces alterations in vital signs, such as increased respiratory and heart rates beyond normal levels [8]. Patients with COVID-19 experience significant increases in respiratory rate and slight increases in heart rate, with no significant increase in body temperature [9]. Therefore, in addition to fever-screening thermography, respiratory and heart rate measurements can be used for the accurate identification of patients with infections.

Recent advances have highlighted the importance of noncontact multi-vital sensing systems, which can simultaneously capture multiple physiological parameters. Hassanpour and Yang [10] comprehensively reviewed the rapid progress in multimodal and multitask approaches, including vision-based, radar-based, and thermal imaging modalities. Notably, a composite-type camera integrating a depth sensor for respiratory rate, an infrared thermal camera for body temperature, and an RGB camera for heart rate has been developed and clinically validated [11]. More recently, a Fusion-Vital framework combining RGB video and radio-frequency signals has been proposed to estimate both respiration and heartbeat, illustrating the potential of multimodal fusion to overcome the limitations of single-modality systems [12]. These studies collectively underscore the importance of multimodal integration for robust, noncontact monitoring.

Several combinations of infrared thermography and other remote sensing techniques have been proposed for screening for infectious diseases. The temperature of expired air is higher than that of inspired air owing to heat exchange in the lungs and respiratory passageways [13]. Therefore, infrared thermal cameras can be used to evaluate respiratory rates in a contactless manner by detecting temperature fluctuations around the nostrils during the respiratory cycle. Negishi et al. [14] developed a remote multi-vital sensing system to screen for patients with an infection. This system combines an infrared camera to measure the temperature and respiratory rate and a video camera to measure the heart rate. The body temperature and temperature changes around the nasal cavities, which indicate breathing, are measured with the face looking straight ahead. The requirement for this position may complicate detection and measurement around the nasal cavities, depending on the physical characteristics of the individual. Other studies have used infrared thermal imaging to monitor respiration by measuring the temperature of the nostrils from an angle lower than that of the front of the face, thereby capturing the entire nasal cavity [15,16,17]. However, these imaging methods cannot simultaneously evaluate body temperature and respiratory rate.

Visualizing the breathing process helps identify respiratory disorders and assess the respiratory rate using images without requiring contact with the individual. Thus far, a mid-wave infrared (MWIR) thermal camera has been used to measure small temperature changes that accompany neural activity in a rat brain [18]. MWIR detectors provide higher sensitivity and lower noise than uncooled long-wave infrared (LWIR) microbolometers [19]. The MWIR thermal camera detects infrared radiation within the 3–5 μm range and converts it to the corresponding temperature values, yielding a two-dimensional temperature distribution map. Given that the MWIR waveband includes an absorption band for carbon dioxide (CO_2_) at approximately 4.3 μm and that expired human air is characterized by high concentrations of CO_2_, this infrared thermal imaging technique can be used for remote imaging of human breathing. This imaging technique has been successful in measuring the respiratory rate [20,21]. However, in those studies, the researchers used an optical bandpass filter tuned to the CO_2_ absorption band in conjunction with the image camera. In addition, this method requires a background, such as a wall, to create a high contrast with CO_2_. Therefore, the measurements were limited to capturing the face in the profile. Moreover, facial thermal imaging from a lateral perspective poses challenges in evaluating body temperature according to the guidelines for thermography measurements of the human body temperature at the inner canthus [22,23]. Nonetheless, imaging-based detection of CO_2_ from the frontal face could enable the simultaneous evaluation of body temperature and respiratory rate using an infrared thermal camera.

Techniques for the remote detection of heart rate using facial videos have been developed. These techniques rely primarily on subtle color changes in the human skin caused by light absorption by blood. The multimodal clinical screening system used in the previous study [14] comprised a thermal image sensor for body temperature and respiratory rate and a color complementary metal-oxide semiconductor sensor for heart rate. Additionally, infrared thermal cameras have been used for contactless pulse measurements. This method of temperature monitoring is based on the fact that temperature modulation due to pulsating blood flow produces a thermal signal in the superficial vessel [24]. In the previous study [24], the external carotid or the superficial temporal arteriovenous complex of the face in profile images was selected to evaluate the heart rate. However, the supraorbital vessels were selected for some subjects. Therefore, in addition to estimating the body temperature using a thermal camera, the heart rate can be evaluated using full-face thermal images.

This study introduces a multi-vital remote sensing technique using an MWIR thermal camera, aiming to develop more powerful systems for infection screening and smart vital sensing that can be used in domestic settings. First, the respiratory rate was evaluated in a contactless manner by visualizing the expired air in a frontal face image captured using an MWIR thermal camera, leveraging the infrared absorption by CO_2_. Next, the body temperature and heart rate were estimated using the same thermal images of the frontal face after processing.

## 2. Materials and Methods

### 2.1. Subjects

This study was approved by the Ethics Committee of the Center for Health Science Innovation, Osaka City University (approval no. 41, granted on 30 June 2021). All participants signed an informed consent form before enrolling in the study. The participants comprised six healthy male adults (average age of 43.3 ± 3.1 years, body mass of 64.2 ± 11.5 kg, and height of 1.7 ± 0.05 m). The experiments were conducted in a temperature-controlled room equipped with an air conditioner (approximately 24 °C and 50% relative humidity).

### 2.2. Setup

**Figure 1:**
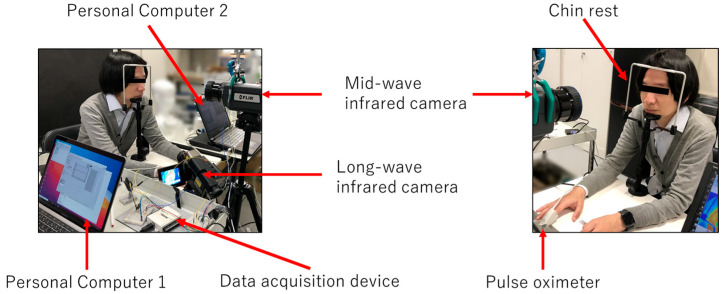
Experimental setup of the proposed multi-vital remote-sensing system. The system comprises a mid-wave infrared camera, a long-wave infrared camera, a pulse oximeter, a data acquisition device, and two personal computers. The subject’s face is fixed against a chin rest to ensure stable positioning during measurement.

Figure 1 shows the setup of the proposed multi-vital remote-sensing system. The subjects were each seated on a chair in front of an MWIR camera (FLIR A6700s, FLIR Systems, Wilsonville, OR, USA), which is sensitive to thermal energy in the range of 3–5 μm, at a distance of approximately 80 cm. During the measurement, with their faces fixed against a chin rest (Medium Duty Chin Rest, BIOFIELD, Inc., Osaka, Japan), the subjects were asked to remain as motionless as possible, limiting unnecessary body movements, and breathe with their mouths closed. Each subject faced the MWIR camera and breathed spontaneously while the camera was recording. A pulse oximeter (Pulse Lab, IWS920-DEV, Tokyo Devices, Inc., Tsukuba, Japan) was attached to the left middle finger of each subject. An additional infrared thermal camera (InfRec R550 pro, Nippon Avionics Co., Ltd., Osaka, Japan), which is sensitive to LWIR thermal energy in the range from 8 to 14 μm, was placed on the left side of the MWIR camera to provide a reference for respiration. Because the LWIR camera measured the temperature fluctuations in the nasal cavities of the subject, it was mounted toward the subject from a position below the face level to ensure that the nostrils were completely visible. These cameras were triggered using the LabVIEW software (ver. 2020, National Instruments Corp., USA), which in turn triggered the recording in each camera software (FLIR Research IR max 4.4 for the MWIR camera and InfReC Analyzer NS9500 Professional for the LWIR camera).

### 2.3. Protocol

Each subject performed spontaneous breathing for 40 s utilizing the previously described experimental setup. Each subject performed five trials.

### 2.4. Data Acquisition

The MWIR camera recorded at a speed of 60 frames per second (fps) with a pixel resolution of 640 × 512 pixels, whereas the LWIR thermal camera recorded at a speed of 30 fps with a pixel resolution of 640 × 480 pixels. The MWIR thermal images were transferred using a standard GigE Vision^®^ interface from the camera to a personal computer and captured using the ResearchIR software version 4.40.6.24 (FLIR systems Inc., Wilsonville, OR, USA). The acquired thermal images were converted to temperature values and saved in TIFF format by the software. The LWIR thermal camera was connected to the same personal computer via USB 2.0, and the images were captured by camera control software (InfRec Analyzer NS9500 Professional version 7.1C, Nippon Avionics Co., Ltd., Japan) installed on the computer. The images were saved in JPEG format, and the temperature values were saved as text data. Image capture was initiated when each camera received a trigger pulse generated from a USB multifunction I/O device (USB-6009, National Instruments Corp., Austin, TX, USA) controlled using in-house software written in LabVIEW 2020 (National Instruments Corp., Austin, TX, USA). The acquisition of image data was synchronized with the start of the pulse oximeter recording. The pulse oximeter recorded photoplethysmography signals at 409.6 Hz. These images were saved on a computer by each camera control software, whereas the pulse signals were saved on another computer using in-house software written in LabVIEW. Before the experiments, the body temperature of each subject was measured at the axilla, tympanic membrane, and forehead. The axillary temperatures were measured using digital thermistor probes (MC-171W and MC-687; Omron Healthcare Inc., Kyoto, Japan) in two different modes: actual (direct) measurement (MC-171W) and predictive measurement (MC-687). The subjects were each asked to place the probe on the axilla until the temperature was stable, which usually took approximately 10 min in the actual mode and approximately 15 s in the predictive mode. The actual measurement was performed once, whereas the predictive measurement was performed three times. The accuracy and temperature resolution were ±0.1 and 0.1 °C, respectively.

### 2.5. Data Analysis

#### 2.5.1. Visualization of Respiration

**Figure 2:**
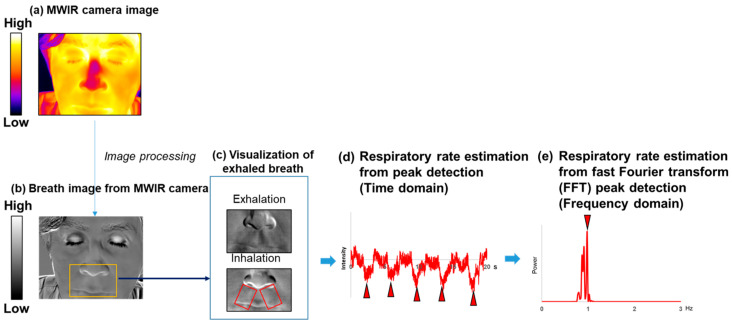
Overview of breath visualization and respiratory rate measurement using mid-wave infrared (MWIR) camera. (**a**) Example of a MWIR camera image; (**b**) processed image visualizing exhaled airflow, with the yellow square marking the region of interest; (**c**) cropped images from the yellow square shown in (**b**), showing an example of exhalation and inhalation images. Red rectangles in (**c**) indicate the regions of interest (ROIs) used to measure brightness changes associated with visualized exhaled air; (**d**) example of a respiratory signal obtained from exhalation visualization images; (**e**) fast Fourier transform of the signal shown in (**d**). The red triangle indicates the peak location.

In the MWIR waveband, the infrared absorption by CO_2_ creates an image contrast, enabling the visualization of CO_2_ as dark regions in thermal images (Figure 2). To enhance the contrast corresponding to expired CO_2_ and visualize the expiration in the MWIR thermal images, the average intensity image of a time-stack image was subtracted from each individual image within the stack using Fiji (ImageJ version 1.52n; ImageJ2 version 2.0.0-rc-43; Java1.6.0_24 [64bit]) [25].

#### 2.5.2. Respiratory Rate

**Figure 3:**
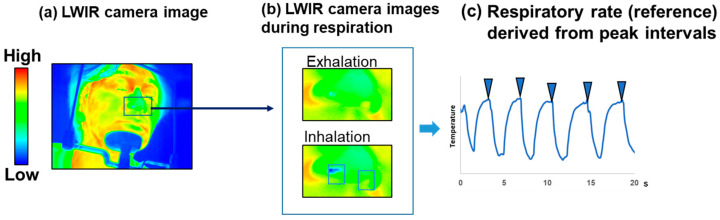
Overview of respiratory rate measurement using a long-wave infrared (LWIR) camera. (**a**) Example of an LWIR camera image with a black square marking the region of interest; (**b**) cropped images from the blue square shown in (**a**), showing an example of exhalation and inhalation images. Light blue rectangles in (**b**) indicate the ROIs placed above each nostril to measure the mean temperature; (**c**) example of a respiratory signal derived from the images shown in (**b**). The blue inverted triangles indicate peak locations.

A respiratory curve was derived from the MWIR images (Figure 2) to observe temporal respiratory changes. In each MWIR image, a rectangular region of interest (ROI) was placed over the visualized exhalation, which manifested at a lower intensity. In each LWIR image, a rectangular ROI delineated each nasal cavity (Figure 3). The temporal changes were calculated using a plot of the *z*-axis profile function in Fiji. The method employed to monitor respiration by detecting temperature changes across the nostrils using an IR camera is described in a previously published paper [26]. Therefore, the respiratory curves derived from the LWIR camera images were accepted as reference data for respiration. In this study, the average values of the respiratory signals from both nostrils were used. The respiratory rate was estimated by passing the temporal change signals through a second-order Butterworth digital bandpass filter, centering the passband at the respiratory rate frequency (0.1–0.4 Hz). The peak detector virtual instrument (VI) function in LabVIEW was used to calculate the interval times from peak to peak and to determine the respiratory rate [27]. All intervals were averaged, and the reciprocal of the averaged value was multiplied by 60 to convert it to the respiratory rate in breaths per minute (breaths/min) (time domain). An alternative method for estimating the respiratory rate consists of using a fast Fourier transform (FFT). The filtered signals were converted to the frequency domain using FFT, and the spectral peak was determined to calculate the respiratory rate. The spectral measurement VI in LabVIEW [28] was also employed. These data analyses were performed using in-house software written in LabVIEW (frequency domain).

#### 2.5.3. Body Temperature Evaluation Using the IR Camera

**Figure 4:**
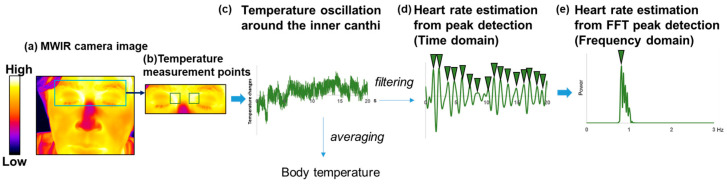
Overview of body temperature evaluation and heart rate measurement using an MWIR camera. (**a**) Example of an MWIR image with a blue square marking the region of interest; (**b**) cropped image from the blue square shown in (**b**), showing two green squares at the inner canthus that indicate the sites of temperature measurement; (**c**) example of a temporal temperature signal obtained from the regions outlined in (**b**); (**d**) fast Fourier transform of the signal presented in (**c**). The green inverted triangles indicate peak locations.

The procedure and measurement sites for the temperature measurement method used to identify humans with fever via thermography are described in the technical report ISO/TR 13154:2017 [22]. The report recommends focusing on the inner canthus (tear duct) regions. The skin temperature between the medial aspect of the orbit and the nose was defined as the index for body temperature (Figure 4). Because the measurement areas nearly coincided with the regions of highest temperature on the face, the measurement area was segmented by binarizing the averaged image of a stack using a predetermined temperature threshold. The body temperature was calculated as the average temporal temperature in the area. The binarization and averaging processes were performed using Fiji.

#### 2.5.4. Heart Rate Evaluation Based on IR Camera–Captured Temperature Changes

Noncontact evaluation of the heart rate was performed using a thermal camera, in accordance with methods demonstrated in previous studies [24]. The heart rate was estimated by passing the temporal change signals through a bandpass filter (second-order Butterworth digital filter) such that the passband was centered at the respiratory rate (0.8–1.5 Hz frequency) (Figure 4). The time intervals were calculated from peak to peak to determine the heart rate using the same in-house software written in LabVIEW 2020 (National Instruments Corp., Austin, TX, USA) used for the respiratory rate. All intervals were averaged, and the reciprocal of the average value was multiplied by 60 to convert it into the heart rate in beats per minute (beats/min).

#### 2.5.5. Estimation of Respiratory and Heart Rates from Pulse Oximeter Signals

In accordance with earlier studies [29,30,31], the respiratory and heart rates were estimated by applying bandpass filters to the pulse oximeter signal, isolating the respiratory and cardiac frequency components, respectively. This approach was used to obtain reference values for comparison with the output of the method proposed in this study. Signal processing was performed using custom-built LabVIEW programs, similar to those designed for the digital filtering techniques used to extract vital signs from the thermal images.

### 2.6. Evaluation of Exhaled CO_2_ Volume

#### 2.6.1. Measurement of Nasal Airflow Velocity

**Figure 5:**
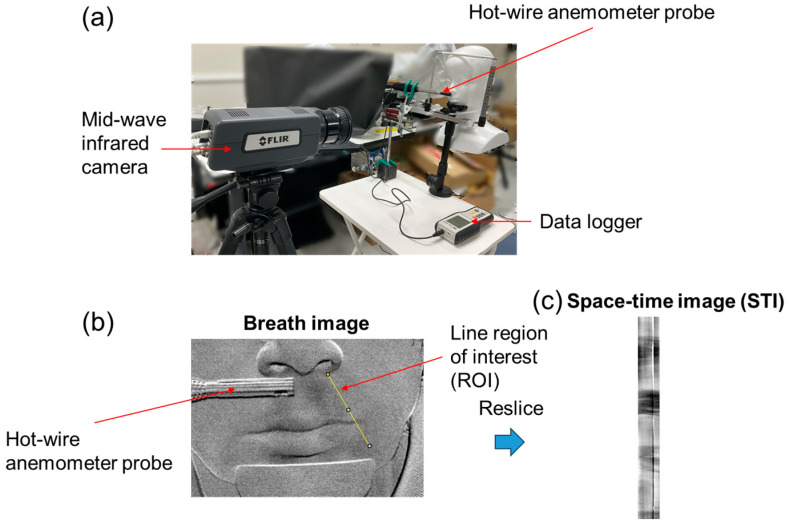
Experiment for measuring exhaled airflow velocity. (**a**) Experimental setup for measuring exhaled flow velocity; (**b**) overview of space–time image (STI) extraction; (**c**) example of STI.

The nasal airflow velocity was measured using a hot-wire anemometer (HT-9829, Dongguan Xintai Instrument Co., Ltd., Dongguan, China) at 1 Hz (Figure 5a). The sensor was positioned at the tip of the anemometer probe below either the left or right nasal cavity to ensure that exhaled airflow passed through it. Thermal images were captured using an MWIR camera to simultaneously visualize the exhaled airflow. The measurements were conducted for each subject. The airflow velocity was measured five times at each nostril, with each measurement requiring 20 s to complete.

#### 2.6.2. Image-Based Estimation of Exhaled Airflow Velocity

**Figure 6:**
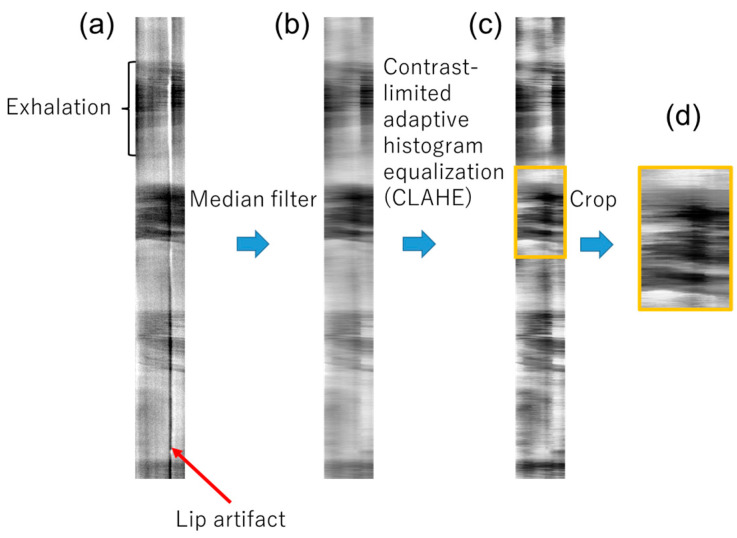
Image processing steps for the STI. (**a**) Example of STI; (**b**) STI after applying a median filter; (**c**) STI after applying contrast-limited adaptive histogram equalization to the image in (**b**); (**d**) cropped image of the exhalation region outlined in (**c**).

**Figure 7:**
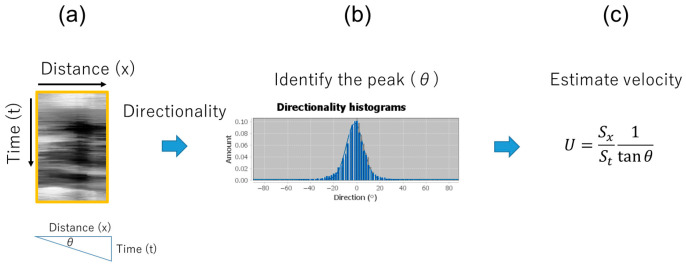
Overview of methods for evaluating flow velocity from the STI. (**a**) Same as Figure 6d, with the horizontal axis representing the distance and the vertical axis representing the time. The relationship between time, distance, and angle *θ* is shown below; (**b**) directionality analysis used to identify peak *θ*; (**c**) formula for calculating the velocity *U* from *θ*.

The spatiotemporal image velocity (STIV) method, which is commonly used to evaluate river surface flow [32,33,34], was employed to estimate exhaled airflow velocities from image sequences. STIV analyzes the spatiotemporal patterns of brightness variations in video frames to extract velocity information, offering a noncontact and efficient approach to flow measurement. First, a linear ROI was set along the direction of the visualized flow. Next, the thermal image was resliced along the ROI using the “Reslice” function of Fiji, resulting in a space–time image (STI) (Figure 5b). Figure 6 shows the image processing steps used for the STI. A median filter (σ = 15) was applied in the x-direction of the STI to reduce noise when the ROI overlapped with the lip. Contrast-limited adaptive histogram equalization was then applied to emphasize the luminance gradient depicted on the STI using a Fiji function. In the contrast-enhanced images, the region corresponding to each exhalation was extracted, and the Fiji analysis plugin, “Directionality” [35], was used to obtain the mean orientation angle (*θ*) of the structure present in the input image (Figure 7). Because the length and time scales of the STI are known, the mean velocity *U* (m/s) can be calculated using the following equation:

(1) $$U=\;\frac{S_{x}}{S_{t}}\frac{1}{\tan\theta }$$

where *S_x_* is the unit length scale along the search line (m/pixel) and *S_t_* is the unit time scale of the time axis (s/pixel), which depends on the frame rate of the camera. However, in reality, exhaled air diffuses from the nasal cavity. This makes the nasal cavity area unsuitable for accurate estimation of the flow volume. Nonetheless, in this study, the fluid was assumed to maintain unidirectional flow from the release surface without diffusion in an ideal state. Therefore, under the ideal-state assumption, the volume of exhaled airflow, *V* (m^3^/s), was estimated by multiplying the airflow velocity by the area of the nasal cavity. This calculation was based on the assumption that CO_2_ moved at the same velocity as that of exhaled breath. Because exhaled CO_2_ constitutes 4% of the total exhaled breath, the exhaled CO_2_ volume *V_CO2_* was estimated as 0.04*V*.

### 2.7. Statistical Analysis

The experimental data are expressed as the mean ± standard deviation. Scatter and Bland–Altman plots were used as statistical and graphical evidence of the agreement between the proposed and reference methods [36]. All analyses related to the evaluation of agreement were conducted using Python (v. 3.13.5) in a Jupyter Notebook environment (v. 7.3.2). Calculations were performed using the Python libraries Pandas (v. 2.2.3), NumPy (v. 2.1.3), and SciPy (v. 1.15.3). Here, the agreement rate was calculated as the proportion of data points falling within the limits of agreement (mean bias ± 1.96 standard deviation) in the Bland–Altman plot.

The linear correlation between the proposed and reference methods was determined using Pearson’s correlation coefficient analysis. The level of significance was set at *p* < 0.05. The normality of each group was assessed using the Shapiro–Wilk test. If both groups demonstrated normality (*p* ≥ 0.05), the equality of variances was tested using Bartlett’s test. If the groups were homoscedastic (equal variances), Student’s *t*-test was applied to compare the group means. If either group failed the normality test or if normality was confirmed but equal variance was not, group differences were evaluated using the Mann–Whitney U test, a nonparametric alternative that does not assume normality or homogeneity of variance. A summary of the normality and variance test results, together with the statistical methods used for each comparison, is provided in Supplemental Table S1.

## 3. Results

### 3.1. Visualization of Respiration

Figure 2 shows a representative result of the breath images. In the processed MWIR thermal images resulting from the proposed method, haze-like shadows darker than the surrounding elements were observed between the nose and upper lip (Supplementary Video S1). Media 1 shows the time sequence during respiration, as revealed by MWIR thermal images that underwent image processing. The supplementary video reveals a shadow indicating flow from the nasal cavities, corresponding to expiration. Figure 3 shows a representative LWIR thermal camera image capturing the same process during respiration. The nasal temperatures increased with exhalation and decreased with inhalation.

**Figure 8:**
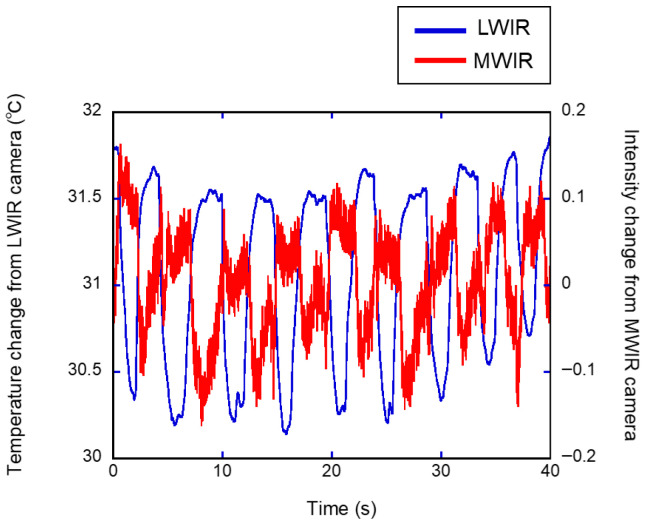
Temporal changes in the nasal cavity temperature captured by the LWIR camera (blue) and image intensity captured by the MWIR camera (red).

**Figure 9:**
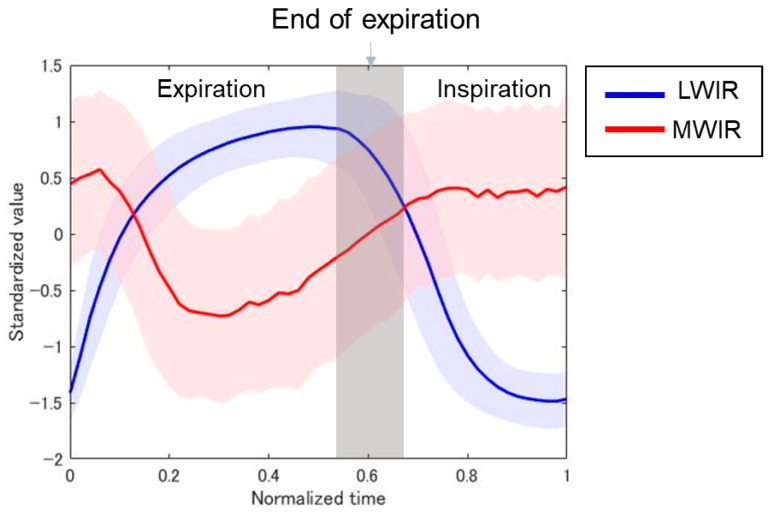
Normalized signal profiles of a single respiratory cycle obtained via LWIR (blue) and MWIR (red) detection. The solid lines represent the mean of several trials, and the shaded bands in lighter hues of the same color (light blue for LWIR and light pink for MWIR) indicate the variability range (±1 standard deviation). The gray region indicates the end of expiration.

Figure 8 shows the time courses of the normalized temperature values obtained from the nostril ROI in the LWIR thermal image and the ROI below the nose in the MWIR thermal image. The nasal temperature changes obtained using the LWIR thermal camera exhibited periodic variations. In contrast, in the visualized breath image obtained using the MWIR camera, the signal intensity change—which contained more high-frequency noise than the LWIR temperature change—decreased with exhalation, showing an inverse phase against the LWIR temperature changes; however, the cycles were largely consistent. Furthermore, for the continuous respiratory signals obtained from all subjects, breath-by-breath segmentation and temporal normalization were performed separately on the data acquired with both the MWIR and LWIR cameras to analyze the average waveform patterns (Figure 9). The respiratory signals obtained using the LWIR camera exhibited patterns similar to those of a typical capnogram. During the expiratory phase, the temperature increased sharply, followed by a gradual increase until it reached a peak. In the subsequent inspiratory phase, the temperature decreased rapidly, then decreased gradually, and ultimately returned to its initial baseline. In the data obtained from the MWIR camera, the brightness value exhibited a sharp decline shortly after the onset of expiration, reaching a minimum value earlier than the peak observed in the LWIR signal. Subsequently, the brightness increased and returned to baseline during the inspiratory phase.

### 3.2. Respiratory Rate

**Figure 10:**
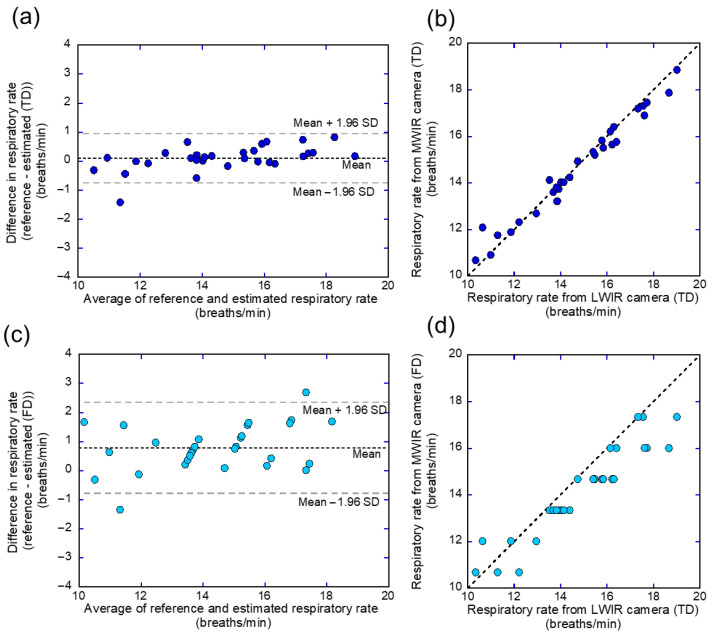
Bland–Altman and scatter plots of the respiratory rate estimated from MWIR images in the time domain (TD) and frequency domain (FD) compared with the LWIR reference. (**a**) Bland–Altman plot of TD-based respiratory rate estimation; (**b**) scatter plot of TD-based respiratory rate estimation; (**c**) Bland–Altman plot of FD-based respiratory rate estimation; (**d**) scatter plot of FD-based respiratory rate estimation. The dotted horizontal lines in (**a**,**c**) indicate the mean differences (bias), and the dashed horizontal lines indicate the limits of agreement (LOA). The dotted lines in (**b**,**d**) represent the identity line.

The respiratory rate was derived from temporal signal fluctuations using both time- and frequency-domain analyses. The respiratory rates estimated from the MWIR thermal signals were compared with those derived from the LWIR-based reference data using time-domain analysis. The agreement between the two methods was assessed using Bland–Altman analysis, which revealed the degree of bias and limits of agreement (LOA) across the measurements. The Bland–Altman plot revealed a mean bias of 0.10 breaths/min between the reference data and the MWIR-based respiratory rate estimated in the time domain, with the LOA ranging from −0.765 to 0.965 breaths/min (Figure 10a). In contrast, the Bland–Altman analysis comparing the reference data and MWIR-based respiratory rate obtained via frequency-domain methods yielded a higher mean bias of 0.786 breaths/min, with a wider LOA ranging from −0.802 to 2.373 breaths/min (Figure 10c).

The agreement rate, calculated as the percentage of data points within the LOA to assess consistency between methods, was 96.6% in the time-domain approach and 93.1% in the frequency-domain approach. Additionally, scatter plots illustrating the relationship between each respiratory rate estimation and the reference data revealed significant positive correlations for both approaches. However, the time-domain method demonstrated a stronger linear association with the reference values, with a correlation coefficient of r = 0.987 and a lower root mean square error (RMSE) of 0.445 breaths/min (Figure 10b). In contrast, the frequency-domain method yielded r = 0.944 with a higher RMSE of 1.118 breaths/min (Figure 10d), indicating lower estimation accuracy. These findings collectively suggest that the time-domain approach offers closer agreement and a more reliable estimation of respiratory rate under the conditions evaluated. A summary of the key quantitative metrics for both methods is presented in Table 1. These results indicate that the time-domain approach yielded closer agreement with the reference, suggesting that it may provide more reliable respiratory rate estimates under the given conditions.

### 3.3. Body Temperature

**Figure 11:**
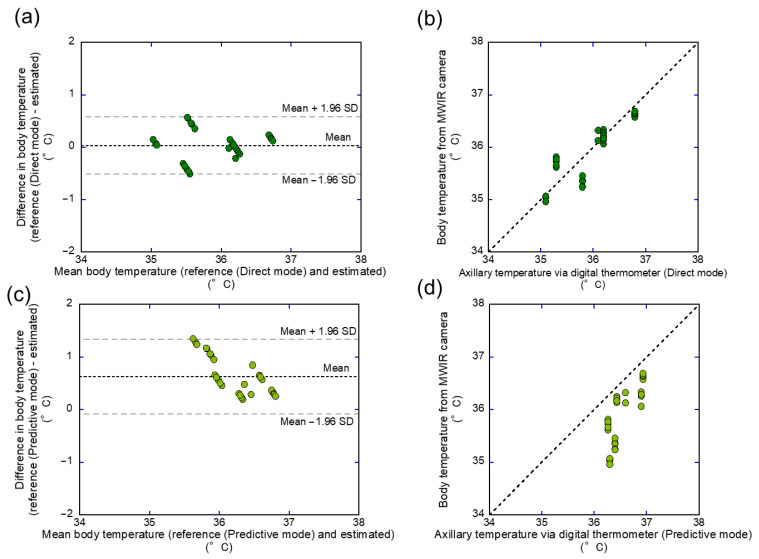
Bland–Altman and scatter plots comparing MWIR-derived body temperature with axillary temperature based on direct and predictive methods. (**a**) Bland–Altman plot comparing MWIR-derived temperature with axillary direct temperature; (**b**) scatter plot comparing MWIR-derived temperature with axillary direct temperature; (**c**) Bland–Altman plot comparing MWIR-derived temperature with axillary predictive temperature; (**d**) scatter plot comparing MWIR-derived temperature with axillary predictive temperature. The dotted horizontal lines in (**a**,**c**) indicate the mean differences (bias), and the dashed horizontal lines indicate the LOA. The dotted lines in (**b**,**d**) represent the identity line.

Body temperature was evaluated using MWIR thermal images and compared with reference thermometer readings. The axillary temperature was measured in both direct and predictive modes. Figure 11 shows Bland–Altman plots and correlation graphs for both direct (a and b) and predictive (c and d) axillary measurements, revealing minimal bias and strong agreement with the camera-derived temperatures. For the direct mode, the Bland–Altman plot showed a bias of 0.036 °C and LOA ranging from −0.522 to 0.594 °C, with a 100% agreement rate. The correlation coefficient was r = 0.864, with an RMSE of 0.282 °C. In contrast, the predictive mode yielded a lower correlation coefficient r = 0.771 and a higher RMSE of 0.728 °C. The Bland–Altman plot indicated a bias of 0.632 °C and a wider LOA ranging from −0.091 to 1.354 °C, although the agreement rate remained 100%. Table 2 summarizes all quantitative metrics, including the correlation coefficients, agreement rates, and RMSE values across the measurement modes and anatomical sites. In addition, the predictive mode consistently yielded higher axillary temperatures compared to the MWIR measurements, and a significant difference in the mean values was observed (Supplemental Table S1). A comparison between the predictive and direct thermometer modes is summarized in Supplemental Table S2. As expected, the agreement with the reference values was generally high, particularly for the direct-mode axillary readings. These results suggest that MWIR camera temperature measurements align more closely with the axillary temperature in the direct mode than in the predictive mode.

### 3.4. Extraction of Pulse Signals

**Figure 12:**
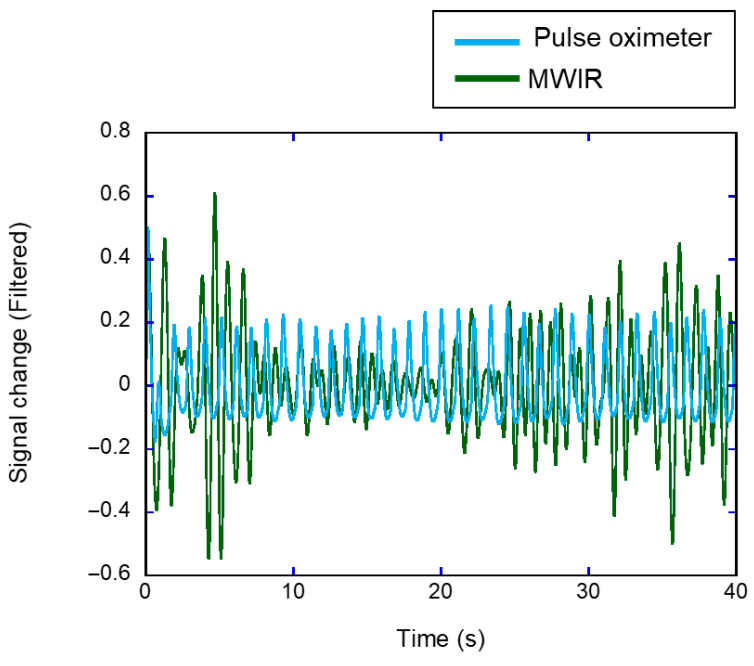
Temporal changes in heartbeat signals derived from pulse oximeter (light blue) and MWIR temperature (green) measurements.

The pulse signals were evaluated using an MWIR thermal camera. Figure 12 shows a filtered signal obtained using the thermal camera overlaid on a reference signal obtained using the pulse oximeter. The signal was processed using a bandpass filter (0.8–1.5 Hz) to remove the high-frequency noise embedded in the time-series body temperature signal (Figure 4). The two waveforms exhibited strong temporal alignment with consistent phase matching and amplitude patterns across multiple cycles. Although the two waveforms exhibited strong temporal alignment with consistent phase matching across multiple cycles, a noticeable difference in amplitude was observed between the MWIR-derived signal and the pulse oximeter reference.

### 3.5. Heart Rate

**Figure 13:**
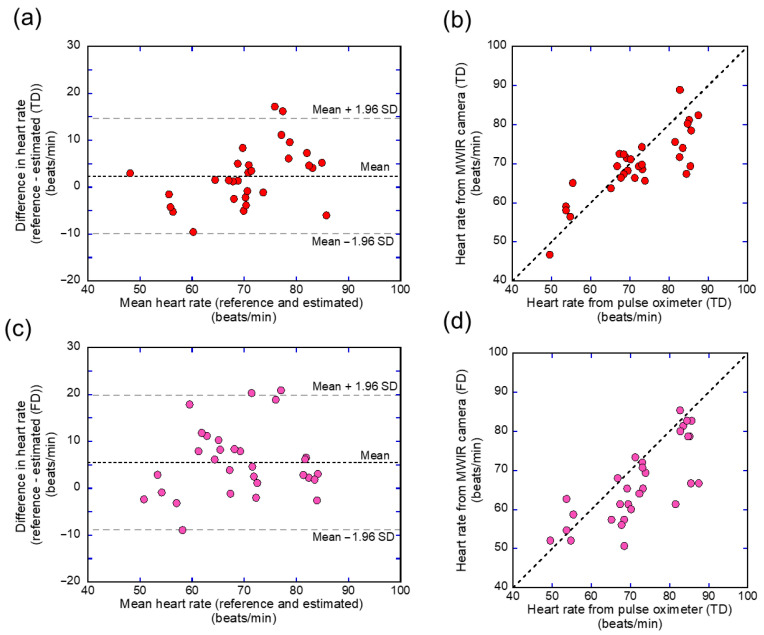
Bland–Altman and scatter plots comparing heart rate (HR) estimates from MWIR signals (TD) and FD) with pulse oximeter reference. (**a**) Bland–Altman plot comparing MWIR TD-based HR with pulse oximeter reference; (**b**) scatter plot comparing MWIR TD-based HR with the pulse oximeter reference; (**c**) Bland–Altman plot comparing MWIR FD-based HR with pulse oximeter reference; (**d**) scatter plot comparing MWIR FD-based HR with the pulse oximeter reference. The dotted horizontal lines in (**a**,**c**) indicate the mean differences (bias), and the dashed horizontal lines indicate the LOA. The dotted lines in (**b**,**d**) represent the identity line.

The heart rate was evaluated by analyzing the pulse waveforms using both time- and frequency-domain analyses. To assess the heart rate, thermal signals acquired in the MWIR range were processed and compared with reference data captured in the LWIR spectrum using time-domain techniques. The degree of agreement between the reference and proposed methods (time and frequency domains) was examined using Bland–Altman plots, revealing systematic bias and variation across measurements (Figure 13).

According to the Bland–Altman analysis, the time-domain estimation of the MWIR-based heart rate had a mean deviation of 2.79 beats/min from the reference, with LOAs ranging from −8.854 to 14.443 beats/min (Figure 13a). In contrast, the Bland–Altman analysis of the frequency-domain MWIR-based heart rate revealed a greater bias of 5.818 beats/min, with broader LOAs ranging from −8.342 to 19.966 beats/min (Figure 13b). Among the two methods, the time-domain approach demonstrated a higher agreement rate (93.1%) than its frequency-domain counterpart (89.7%), based on the proportion of samples within the LOAs. Both approaches demonstrated a significant correlation between heart rate estimates and reference data. The time-domain method yielded a higher correlation (r = 0.831) and lower error (RMSE = 6.474 beats/min) (Figure 13c) than the frequency-domain method (r = 0.761; RMSE = 9.172 beats/min) (Figure 13d). Table 3 presents the numerical comparisons between the two methods. Collectively, the results indicate that the time-domain method provides more precise and dependable heart rate estimates than the frequency-domain method within the tested framework. However, the Bland–Altman analysis revealed a wide LOA, which remains excessively broad for clinical or consumer-grade applications. In addition, a significant difference in the mean values was observed for the frequency-domain estimates compared to the reference heart rate (Supplemental Table S1).

### 3.6. Image-Based Evaluation of Expiratory Flow Velocity

The respiratory airflow velocity was estimated from the MWIR thermal images using the STIV technique. The luminance gradient tensor method was applied to compute the slopes of the STIV images. This approach provides alternative estimations of the flow velocity.

The luminance gradient tensor–based STIV was used to evaluate the airflow in the right and left nostrils separately and to determine the average velocity across both sides. Reference data were acquired using a hot-wire anemometer. The mean velocities and standard deviations are listed in Table 4.

The average bilateral flow velocities were 1.099 ± 0.569 m/s for the anemometer and 1.032 ± 0.346 m/s for the luminance gradient tensor method. Statistical testing using the Mann–Whitney U test revealed no significant difference between the luminance gradient tensor and reference (*p* = 0.7037).

### 3.7. Image-Based Evaluation of Expiratory Flow Volume

The expiration flow volume for each nostril was estimated by multiplying the flow velocity, obtained using the luminance gradient tensor method, by the corresponding cross-sectional area. The nasal cross-sectional areas, calculated from a video camera image based on pixel-level spatial calibration, were 71.753 mm^2^ (7.18 × 10^−5^ m^2^) for the right nostril and 58.468 mm^2^ (5.85 × 10^−5^ m^2^) for the left. Under the assumption of constant and non-diffusive airflow (see the Section 2), the estimated flow rates were (7.052 ± 1.687) × 10^−5^ m^3^/s (right) and (6.158 ± 2.332) × 10^−5^ m^3^/s (left). Considering exhalation as a mixed gas, with CO_2_ constituting approximately 4% of the exhalation breath, the CO_2_ emissions were estimated at (2.821 ± 0.6746) × 10^−6^ m^3^/s (right) and (2.460 ± 0.933) × 10^−6^ m^3^/s (left). Therefore, the combined flow volume was (5.281 ± 1.151) × 10^−6^ m^3^/s.

## 4. Discussion

To address the challenge of noninvasive physiological monitoring, this study proposed a multi-vital sensing technique employing a single MWIR thermal camera, with respiration visualization as a key component. In addition to imaging expiratory airflow, this camera enabled simultaneous evaluation of the respiratory rate, heart rate, and body temperature through integrated image and data processing. Furthermore, this study explored the potential for estimating the expiratory flow rate and exhaled CO_2_ volume from respiratory image data. This technique holds promise for high-precision screening of infectious diseases, visual diagnosis of respiratory disorders, and routine health assessments.

Human respiration involves the exhalation of air enriched with CO_2_ and the inhalation of oxygen (O_2_) to maintain vital functions. Monitoring breath dynamics plays an important role in assessing physiological status and detecting signs of respiratory dysfunction and broader physiological abnormalities. In particular, visualizing expiratory airflow can noninvasively offer insights into breathing behavior and gas exchange patterns, forming the basis for diagnostic and screening applications. In this study, an MWIR thermal camera was used to visualize exhaled breath airflow based on the IR absorption of CO_2_.

An MWIR camera containing an indium antimonide (InSb) IR sensor is sensitive to IR wavelengths in the 3–5 μm range and can be employed as a noncontact temperature imaging device with a high temperature resolution [19]. MWIR radiation is emitted from the human body in sufficient quantities within the typical body temperature range and can therefore be utilized for noncontact fever screening. Furthermore, this MWIR camera can detect spectral signatures associated with the absorption band of CO_2_ near 4.3 μm. To enhance subtle temporal variations, each frame is subtracted from the mean image computed across the entire stack, resulting in a set of different images. By leveraging the IR absorption characteristics of CO_2_, this approach can successfully visualize exhaled breath using the MWIR camera (Figure 2 and Media 1). In previous studies, breath visualization using an MWIR camera relied on foreground–background contrast, typically achieved by capturing side-view images of exhaled breath against a static background, such as a wall [20,21]. These approaches also employ narrow optical bandpass filters centered around the CO_2_ absorption band to enhance contrast. Moreover, the method proposed in this study can visualize exhaled breath directly from frontal-view thermal recordings by utilizing CO_2_ absorption characteristics and applying a mean subtraction method without relying on background contrast. Facial images captured by an IR camera enable noncontact body temperature assessment in accordance with ISO guidelines [22,23]. This dual capability enables both body temperature assessment and respiration analysis to be performed in a single imaging session. Furthermore, this imaging approach does not require any external optical systems, such as light sources or optical bandpass filters, making it more adaptable to various experimental and clinical environments.

The visualization of exhaled breath enabled direct observation of respiratory dynamics. Exhaled breath appeared as low-intensity regions in the image owing to absorption by CO_2_. Thus, in this study, the temporal variation in the respiratory signal was extracted by tracing the luminance changes (Figure 2). For comparison, the respiratory signal derived from nasal temperature changes using the LWIR camera (Figure 3) was used as a reference. This approach is based on previous studies indicating that nasal thermal variations closely correspond to respiratory activity [37]. The reference signal changes in Figure 8 show a periodic waveform similar to that of a normal respiratory waveform capnography [38,39]. Moreover, the waveform from the MWIR camera exhibits a periodic structure corresponding to the respiratory cycle, although it includes superimposed high-frequency components. The signal is inversely phased relative to the reference signal, indicating that the signal decreases during exhalation and increases during inhalation. To analyze the respiratory cycle in detail, each cycle was individually normalized and averaged. Consequently, the respiratory cycle recorded using the LWIR camera comprised the following phases: upstroke, plateau, downstroke, and baseline (Figure 9). In contrast, the average normalized signal obtained using the MWIR camera (Figure 9) revealed a negative peak during exhalation, followed by a return to baseline. The peak in the MWIR signal nearly corresponded to the transition point between the expiratory uptake and the plateau observed using the LWIR camera. In fact, the dark flows in the visualized image gradually faded after the transition point and returned to their original brightness levels. Therefore, this respiratory visualization could not capture the inhalation phase. Moreover, the endpoint of the exhalation phase, which appeared in the reference signal, could not be detected in the MWIR signal pattern. However, both respiratory signals obtained using the IR cameras, which exhibited periodic behavior, were nearly synchronized. Thus, the respiratory rate could be determined by analyzing the visualized respiratory images and counting the peaks. The respiratory rates estimated based on peak-to-peak intervals (time domain) and peak frequencies (frequency domain) closely matched those derived from the reference signal (Figure 10 and Table 1). Hence, the proposed respiration visualization method can provide respiratory pattern and respiratory rate estimation without requiring contact with the individual being examined. Therefore, this visualization is useful for the early detection of abnormalities in the respiratory system.

According to the guidelines (ISO & IEC 80601-2-56 [22,23]) and previous studies [5,40], the inner canthus is the recommended site for IR body temperature measurement because of its proximity to the core temperature and its consistent thermal characteristics. Therefore, in this study, the temperature of the parts was evaluated using an MWIR thermal camera and compared with the axillary temperature obtained using electronic thermistors in the direct and predictive modes. The axillary temperatures showed good agreement with the inner canthus temperature measured via MWIR thermography (Figure 11 and Table 2). Furthermore, the direct mode demonstrated better consistency with the inner canthus temperature than the predictive mode. As shown in Supplemental Table S2, the predictive mode consistently yielded higher axillary temperatures than the direct mode. This systematic bias is likely attributable to the internal predictive algorithm of the thermometer, which estimates the equilibrium temperature from short measurement intervals and tends to overestimate it compared with direct readings. It has been reported that the axillary temperature correlates well with the tympanic temperature, suggesting that the axillary temperature may be a reliable indicator of the health state of an individual [41]. This agreement with prior findings further supports the reliability of the direct-mode axillary temperature measurement for estimating the core temperature. Conversely, poor agreement has been reported between the LWIR (7.5–13 µm) infrared camera–derived inner canthus temperature and body core temperatures measured using a gastrointestinal pill and an esophageal probe [42]. In this study, the axillary temperature was used as a reference because of the difficulty in directly measuring the core temperature. Unlike earlier studies, this study utilized an InSb sensor operating in the MWIR range, which offers higher sensitivity and temporal resolution. This difference in sensor characteristics may explain the improved agreement between the inner canthus and reference temperatures observed in the results. In numerous healthcare facilities across Japan, axillary thermometers with predictive mode functionality are routinely used in reception areas prior to medical consultations. In this study, the inner canthus temperature obtained via MWIR thermography showed good agreement with the axillary temperature measured in the direct mode, which is considered more accurate. Therefore, an MWIR-based assessment of the inner canthus temperature could serve as a quick and reliable alternative to direct axillary measurements, making triage and infection control more efficient.

The heart rate can be assessed from slight fluctuations in the temperature of the inner canthus using an MWIR camera. Thermal changes in blood vessels are caused by blood perfusion associated with the cardiovascular pulse. Anatomically, the inner canthus is the area where the arterial supply is close to the skin surface [43]. Pulsatile blood flow changes the tissue temperature because of the heat exchange between the vessels and the surrounding tissue. Previous studies have reported heart rate estimation from an IR thermal image of the carotid vessel in the neck [24]. Therefore, the temperature changes would contain information on vascular pulsation. Compared with the frequency-domain method for temperature changes, the time-domain approach provided more accurate and reliable heart rate estimates (Figure 13 and Table 3). This result can be attributed to the frame rate and acquisition time. In this study, the data were sampled at 60 fps for 40 s, providing a temporal resolution of approximately 0.0167 s for accurate peak detection in the time domain. In contrast, the frequency-domain method was limited by a resolution of 0.025 Hz (1.5 bpm), which may have reduced its accuracy. The temperature resolution of the IR thermal camera also affects heart rate estimation. The heart rate estimation using the LWIR camera required preprocessing, such as noise reduction, and yielded poor results owing to insufficient thermal sensitivity [44]. An MWIR thermal camera typically uses a cooled sensor and has a higher temperature resolution and lower noise-equivalent temperature difference (NETD) than an LWIR camera [19], which generally uses an uncooled sensor. The temperature resolution of the MWIR camera used in this study was 18 mK. Suzuki et al. [18] reported the visualization of slight increases in brain temperature (approximately 50 mK) associated with brain neural activity using an MWIR camera. The high temperature sensitivity enables the detection of subtle temperature fluctuations associated with the pulsation of blood vessels located directly beneath the skin and provides heart rate data using only a bandpass filter targeting the resting heart rate range. In addition, previous studies have reported noncontact heart rate estimation methods based on skin color variations captured by RGB cameras [14,45]. Unlike IR cameras, which passively measure the radiation of a body by providing information on its temperature, the RGB-based heart rate estimation is vulnerable to poor illumination [46]. This study revealed that a single MWIR camera has the potential to measure the body temperature and respiratory rate and that the heart rate could also be estimated using the same device without the need for an additional RGB camera. However, the Bland–Altman analysis revealed relatively wide LOA, indicating that the current framework does not yet achieve the accuracy required for clinical or consumer-grade applications. Moreover, although the MWIR-derived signals followed a temporal trend similar to that of the pulse oximeter, a noticeable difference in amplitude was observed (Figure 12). This discrepancy may be explained by differences in the signal origin and acquisition parameters: the pulse oximeter generates stable electrical signals sampled at 2.4 ms intervals, whereas MWIR detects subtle temperature fluctuations sampled at 16.7 ms intervals with inherently smaller variations. In addition, applying the same bandpass filter to signals with different sampling rates can accentuate edge effects, leading to larger amplitudes at the boundaries of the MWIR signal. Improving accuracy through advanced signal processing, noise reduction, and direct validation against ECG is essential for establishing the reliability of MWIR-based heart rate estimation for practical use. In addition, statistical analysis revealed a significant difference in the mean values for the frequency-domain estimates compared with the reference heart rate (Supplemental Table S1). This discrepancy suggests that frequency-domain analysis may be more susceptible to short-term fluctuations and noise, which can reduce its accuracy compared to time-domain methods. Addressing these limitations is important for improving the robustness of MWIR-based heart rate estimation.

The images of exhaled airflow captured using the MWIR camera enabled the evaluation of flow velocities and flow volumes. The STIV technique, which has achieved good performance in assessing river surface flow velocity from video images, was employed to evaluate the airflow velocity. Using the STIV method, the flow velocity was extracted from the STIs, which depicted the gradient pattern depending on the airflow speed. The estimated velocities were in close agreement with those measured using a hot-wired anemometer. The flow direction extracted from STIs using the Directionality plugin of Fiji tends to be dominated by the initial phase of expiration, where peak expiratory flow occurs. This is because the early stage of exhalation produces the most distinct dark patterns, which strongly influence the directionality histogram. In parallel, the hot-wired anemometer used in this study exhibited limited temporal resolution, making it more sensitive to the peak region rather than being capable of capturing the full time-varying flow profile. Consequently, both methods are anticipated to yield similar velocity values, likely reflecting the dominance of the peak expiratory flow in the measurement process.

In addition to estimating the flow velocity, the STIV method enables an approximate evaluation of the expiratory flow volume. This was achieved by assuming an idealized flow profile in which the exhaled air traveled in a straight laminar stream from the nostrils. However, in practice, the expiratory flow diffuses upon exiting the nasal cavity and does not exhibit laminar characteristics, particularly during the terminal phase of expiration (Movie 1). Despite these complexities, flow volume estimation was performed under the assumption of ideal flow conditions, which allowed for a simplified yet practical interpretation of the velocity data. The CO_2_ exhalation volume was also evaluated by considering the proportion of CO_2_ in the exhaled breath. The Japanese Industrial Standard JIS A 1406 [47], which specifies methods for measuring indoor ventilation rates based on CO_2_ concentration, reports data on the CO_2_ production rate in exhaled breath. In addition, data from the HASS102 standard, established by the Society of Heating, Air-Conditioning, and Sanitary Engineering of Japan (SHASE), were used [48]. These guidelines provide the CO_2_ exhalation volume in the resting state for an adult male. The CO_2_ exhalation volumes are 0.011 m^3^/h as specified in JIS A1406 and 0.0132 m^3^/h according to HASS102. In comparison, the value estimated in this study was approximately 0.019 m^3^/h, which is higher than the guideline value. Nonetheless, although the difference is not negligible, it may be considered acceptable given the methodological assumptions, as the order of magnitude remained consistent. The value estimated in this study was slightly high for a resting state. However, given that the peak expiratory flow velocity is the dominant factor in the calculation, it may still fall within a reasonable range.

Evaluating the CO_2_ exhalation rate is a valuable approach for estimating metabolic activity. Metabolic assessments are essential for understanding energy consumption, physiological status, and health conditions. However, conventional measurement systems require large equipment and complex procedures, which limit their accessibility and practicality. In contrast, the proposed method using an MWIR camera enables noncontact estimation of CO_2_ output while simultaneously capturing other vital signs. This image-sensing approach has demonstrated potential for high-precision fever screening, remote health monitoring, and in-home health checks.

This study should be considered a preliminary proof-of-concept, rather than a definitive validation of clinical applicability. Several limitations must be acknowledged. First, the sample size was extremely small (N = 6) and homogeneous, comprising only healthy adult males. This restricts the generalizability of the findings and introduces gender and age biases. Second, the estimation of expiratory flow volume relied on simplified assumptions (e.g., laminar flow, constant CO_2_ concentration, and approximated nostril area), which are unlikely to hold in realistic physiological conditions. Therefore, the reported values should be regarded as exploratory rather than quantitative. Third, the experimental setup was constrained to resting conditions, with the participants’ heads fixed and their breathing controlled. Although this configuration simplifies the system and minimizes confounding factors, it does not reflect real-world scenarios in which movement, speech, or environmental perturbations are unavoidable. Fourth, the reference measurements used in this study were limited and did not include clinical gold standards (e.g., ECG for heart rate, capnography for respiration, and core temperature for thermometry). This reduces the clinical interpretability of the results. Finally, motion susceptibility was not analyzed; even slight facial movements, blinking, or talking may deteriorate the accuracy of thermal measurements, and the current results should be considered valid only under static conditions. Future work should therefore expand the sample size and include diverse populations, refine the modeling of expiratory flow volume, and validate the framework against physiological gold standards. In addition, incorporating facial tracking and controlled movement experiments is essential for evaluating robustness under realistic scenarios. Furthermore, extending the monitoring framework to include exercise conditions and special environments is important for assessing its practical applicability beyond resting states. These efforts will be critical for establishing the reliability and practical utility of MWIR-based noncontact vital sign monitoring in clinical and consumer applications.

Given the limited sample size, the present results should be interpreted as preliminary and exploratory rather than confirmatory.

## 5. Conclusions

This study proposes a noncontact method for visualizing respiration and monitoring vital signs using an MWIR camera. The visualization of exhaled breath based on MWIR absorption by CO_2_ enables visual inspection and assessment of the respiratory rate and volume. In addition, the high-temperature resolution of the MWIR camera enables not only body temperature measurement but also reliable estimation of the heart rate. By enabling simultaneous estimation of respiration and other physiological parameters without the use of physical sensors, this approach may facilitate physiological monitoring across a wide range of settings. Moreover, this system can be implemented simply by applying the developed analysis method to a single MWIR camera, without the need for complex instrumentation. Such a system would be useful for clinical screening, remote monitoring, in-home health assessments, and daily life applications.

## Figures and Tables

**Table 1:** Evaluation metrics for time- and frequency-domain respiratory rate estimations using the MWIR camera.

RR from the MWIR Camera	Bias (bpm)	SD (bpm)	LOA (bpm)	Agreement Rate (%)	RMSE (bpm)	Pearson Correlations	*p*-Value
Time Domain	0.100	0.441	[−0.765, 0.965]	96.6	0.445	0.987	<0.001
Frequency Domain	0.786	0.810	[−0.802, 2.373]	93.1	1.118	0.944	<0.001

Abbreviations: RR, respiratory rate; SD, standard deviation; LOA, limits of agreement; RMSE, root mean square error; bpm, breaths per minute. All terms are defined in the main text.

**Table 2:** Evaluation metrics for body temperature comparison: MWIR-derived body temperature vs. axillary temperature measurements (direct and predictive methods).

Body Temperature	Bias (°C)	SD (°C)	LOA (°C)	Agreement Rate (%)	RMSE (°C)	Pearson Correlation	*p*-Value
Direct mode	0.036	0.285	[−0.522, 0.594]	100	0.282	0.864	<0.001
Predictive mode	0.632	0.369	[−0.091, 1.354]	100	0.728	0.771	<0.001

Abbreviations: SD, standard deviation; LOA, limits of agreement; RMSE, root mean square error. All terms are defined in the main text.

**Table 3:** Evaluation metrics for time- and frequency-domain heart rate estimations using the MWIR camera.

HR from the MWIR Camera	Bias (bpm)	SD (bpm)	LOA (bpm)	Agreement Rate (%)	RMSE (bpm)	Pearson Correlation	*p*-Value
Time Domain	2.790	5.943	[−8.854, 14.443]	93.1	6.474	0.831	<0.001
Frequency Domain	5.818	7.221	[−8.432, 19.966]	89.7	9.172	0.761	<0.001

Abbreviations: HR, heart rate; SD, standard deviation; LOA, limits of agreement; RMSE, root mean square error; bpm, beats per minute. All terms are defined in the main text.

**Table 4:** Comparison of expiratory flow velocities estimated using an anemometer and the STIV method.

	Right		Left		Average	
	Mean	SD	Mean	SD	Mean	SD
Anemometer (m/s)	1.207	0.666	0.969	0.409	1.099	0.569
STIV (m/s)	1.206	0.288	0.858	0.325	1.032	0.346

Abbreviations: STIV, spatiotemporal image velocity; SD, standard deviation.

## Data Availability

The data are not publicly available due to commercial restrictions related to proprietary imaging equipment and ethical considerations involving human subjects.

## Supplementary Materials

The following supporting information can be downloaded at: https://www.mdpi.com/article/10.3390/s26010098/s1, Video S1: Visualization of exhaled airflow using a mid-wave infrared (MWIR) camera, Table S1: Summary of normality, variance, and statistical test results for each pair, Table S2: Comparison of body temperature measured in direct and predictive modes.
