# Supplementary material for: Noncontact Visualization of Respiration and Vital Sign Monitoring Using a Single Mid-Wave Infrared Thermal Camera: Preliminary Proof-of-Concept
Source: Sensors (Basel). 2025 Dec 23;26(1):98. doi: 10.3390/s26010098 (PMC12787706; doi:10.3390/s26010098)
Supplement: Supplementary file 1 [file sensors-26-00098-s001.zip › sensors-4013514-supplementary/Supplementary Table S1.pdf]

Supplementary Table S1. Summary of normality, variance, and statistical test results for each pair

	Shapiro-Wilk Test (p-value)	Normality Assumption	Bartlett's Test (p-value)	Equal Variance	Statistical Test Applied (p-value)
**RR reference vs estimated (TD)**	0.029	Not normal	-	-	Mann-Whitney U test 0.803
**RR reference vs estimated (FD)**	0.803	Normal	0.503	Equal variance	Student's t-test 0.194
**Body temperature Direct mode**	0.216	Normal	0.852	Equal variance	Student's t-test 0.802
**Body temperature Predictive mode**	0.025	Not normal	-	-	Mann-Whitney U test $p < 0.0001$
**HR reference vs estimated (TD)**	0.784	Normal	0.221	Equal variance	Student's t-test 0.273
**HR reference vs estimated (FD)**	0.265	Normal	0.821	Equal variance	Student's t-test 0.038
**Anemometer vs STIV**	-	Not normal	-	-	Mann-Whitney U test 0.704

Abbreviations: RR, respiratory rate; TD, time-domain; FD, frequency domain; HR, heart rate; STIV, spatiotemporal image velocity; SD, standard deviation. For Anemometer vs STIV, Shapiro-Wilk test could not be applied due to sample size constraints; therefore, Mann-Whitney U test was directly used.
