# Supplementary material for: Noncontact Visualization of Respiration and Vital Sign Monitoring Using a Single Mid-Wave Infrared Thermal Camera: Preliminary Proof-of-Concept
Source: Sensors (Basel). 2025 Dec 23;26(1):98. doi: 10.3390/s26010098 (PMC12787706; doi:10.3390/s26010098)
Supplement: Supplementary file 1 [file sensors-26-00098-s001.zip › sensors-4013514-supplementary/Supplementary Table S2.pdf]

Supplementary Table S2. Comparison of body temperature measured in direct and predictive modes

**Thermometer**	**Predict mode**		**Direct mode**		**Difference**	
Subject	Mean	SD	Mean	SD	Mean	SD
**1**	36.5	0.12	35.6	0.32	+0.85	0.28
**2**	36.7	0.16	35.9	0.20	+0.76	0.16
**3**	36.5	0.23	35.9	0.246	+0.60	0.16

Abbreviations: SD, standard deviation. Subject 1 and 2 were male, and Subject 3 was female.
